# Randomized trial comparing suture button with single 3.5 mm syndesmotic screw for ankle syndesmosis injury: similar results at 2 years

**DOI:** 10.1080/17453674.2020.1818175

**Published:** 2020-09-10

**Authors:** Benedikte Wendt Ræder, Ingrid Kvello Stake, Jan Erik Madsen, Frede Frihagen, Silje Berild Jacobsen, Mette Renate Andersen, Wender Figved

**Affiliations:** a Department of Orthopaedic Surgery, Baerum Hospital, Vestre Viken Hospital Trust; b Kalnes Hospital, Østfold Hospital Trust; c Division of Orthopaedic Surgery, Oslo University Hospital; d Institute of Clinical Medicine, Faculty of Medicine, University of Oslo; e Department of Radiology and Nuclear Medicine, Oslo University Hospital, Oslo; f Aleris Hospital, Tromsø, Norway

## Abstract

Background and purpose — Better outcomes are reported for suture button (SB) compared with syndesmotic screws (SS) in patients treated for an acute ankle syndesmotic injury. One reason could be that screws are more rigid than an SB. A single tricortical 3.5 mm syndesmotic screw (TS) is the most dynamic screw option. Our hypothesis is that 1 SB and 1 TS provide similar results. Therefore, in randomized controlled trial, we compared the results between SB and TS for syndesmotic stabilization in patients with acute syndesmosis injury.

Patients and methods — 113 patients with acute syndesmotic injury were randomized to SB (n = 55) or TS (n = 58). The American Orthopedic Foot & Ankle Society (AOFAS) Ankle–Hindfoot Score was the primary outcome measure. Secondary outcome measures included Manchester Oxford Foot Questionnaire (MOXFQ), Olerud–Molander Ankle score (OMA), visual analogue scale (VAS), EuroQol- 5D (EQ-5D), radiologic results, range of motion, complications, and reoperations (no implants were routinely removed). CT scans of both ankles were obtained after surgery, and after 1 and 2 years.

Results — The 2-year follow-up rate was 84%. At 2 years, median AOFAS score was 97 in both groups (IQR SB 87–100, IQR TS 90–100, p = 0.7), median MOXFQ index was 5 in the SB group and 3 in the TS group (IQR 0–18 vs. 0–8, p = 0.2), and median OMA score was 90 in the SB group and 100 in the TS group (IQR 75–100 vs. 83–100, p = 0.2). The syndesmotic reduction was similar 2 years after surgery; 19/55 patients in the SB group and 13/58 in the TS group had a difference in anterior syndesmotic width ≥ 2 mm (p = 0.3). 0 patients in the SB group and 5 patients in the TS group had complete tibiofibular synostosis (p = 0.03). At 2 years, 10 TS were broken. Complications and reoperations were similar between the groups.

Interpretation — We found no clinically relevant differences regarding outcome scores between the groups. TS is an inexpensive alternative to SB.

Since 2018, several meta-analyses have been published evaluating treatment of acute ankle syndesmotic injury, reporting better outcomes for suture button (SB) fixation compared with syndesmotic screw (SS) (Shimozono et al. 2018, McKenzie et al. 2019). Shimozono concluded that the SB technique resulted in improved outcome and lower rates of joint malreduction. These results are based on heterogenous studies: different fracture types were compared; different numbers of implants were used and different diameters and cortices were engaged for SS fixation (Shimozono et al. 2018). Andersen et al. (2018) reported superior results for SB compared with a quadricortical 4.5 mm SS. A quadricortical SS necessitates routine screw removal, with a 5–9% reported risk of wound infection (Schepers et al. 2011, Andersen et al. 2015) and potential loss of reduction after implant removal (Laflamme et al. 2015). A quadricortical SS is a rigid fixation, inhibiting tibiofibular movement throughout the gait cycle (Riedel et al. 2017, Ramsey et al. 2018). The SB has a higher implant cost compared with SS (Ramsey et al. 2018), may not be sufficient to maintain fibular length in Maisonneuve fractures (Riedel et al. 2017), and has an implant removal rate of 6%, mainly due to irritation from the lateral knot (Andersen et al. 2018). The single tricortical 3.5 mm syndesmotic screw (TS) allows for some tibiofibular movement (Clanton et al. 2017), making the TS an inexpensive alternative, without need for routine implant removal. In this study we compare outcomes between a knotless SB and TS. Our hypothesis was that there is no difference in outcomes in patients treated with SB and a 3.5 mm TS.

## Patients and methods

### Patients and procedures

**Figure 1.**
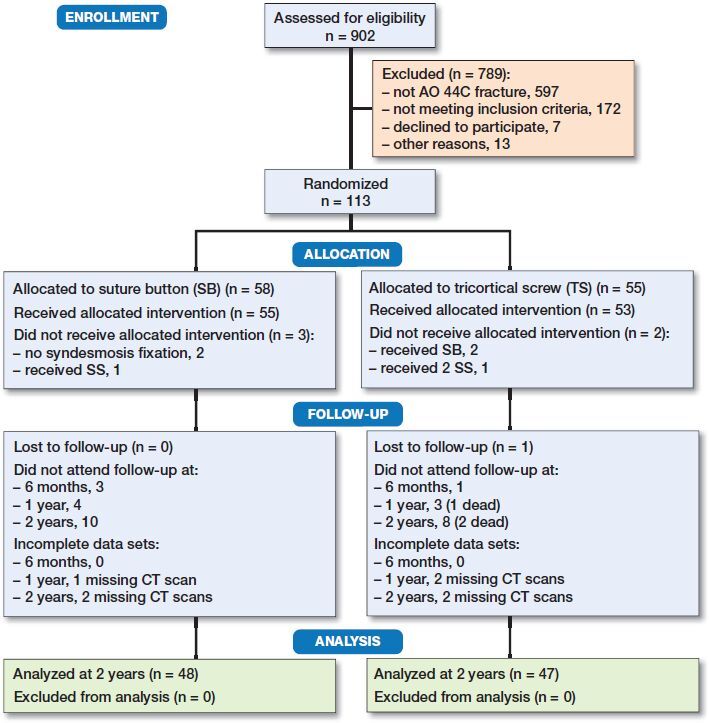
CONSORT flowchart of the trial enrollment and analysis.

3 hospitals participated in recruiting and treating patients. Surgery was conducted by 45 different surgeons. Patients were included by the orthopedic resident on call, from January 2016 to September 2017. Patients aged 18 to 69 who had suffered an acute AO type 44-C ankle fracture assessed by radiographs were asked to participate (Figure 1). Exclusion criteria were polytrauma, open fractures, previous fracture or arthritis of the same ankle, or neurologic impairment of the lower limbs. A web-based randomization system was used, developed and administered by Clinical Research Unit Central Norway, Norwegian University of Science and Technology, Trondheim, Norway.

Surgery was performed according to AO principles. The syndesmosis was reduced and fixed in a closed manner, guided by fluoroscopy. Surgeons were recommended to fix the syndesmosis at a level just proximal to the inferior tibiofibular joint (Barbosa et al. 2020), the use of temporary fixators (K-wire or reduction clamp) was decided by the surgeon. Patients allocated to SB were treated with a single knotless SB (Ziptight, Zimmer Biomet, Warsaw, IN, USA). Patients allocated to TS were treated with a fully threaded self-tapping, 3.5 mm tricortical screw (DePuy Synthes, West Chester, PA, USA). The screw length was not specified, but standardized to engage 3 cortices. Surgery was performed by the on-call team, either by an experienced resident, or a less experienced resident accompanied by a consultant or senior resident. Antibiotic prophylaxis was given as a single dose peroperatively. All patients followed the same protocol postoperatively: implants were not routinely removed; plaster casts and thrombosis prophylaxis were not used routinely. Patients were advised partial weight-bearing (20–30 kg) directly after surgery (Barbosa et al. 2020), then weight-bearing as tolerated after 6 weeks.

### Outcome measures

Patients were assessed by an orthopedic surgeon and a physiotherapist at 6 weeks, 6 months, 1 and 2 years. The physiotherapists who conducted the physical examinations were blinded to the treatment allocation. The main outcome measure was the American Orthopedic Foot & Ankle Society (AOFAS) Ankle–Hindfoot scale. AOFAS incorporates subjective and objective factors into a numerical scale of 0 to 100, 100 being the best. Secondary outcome measures included the Manchester Oxford Foot Questionnaire (Dawson et al. 2007, 2011) (MOXFQ), a 16-item (each item scored 0–4) patient reported outcome measure (PROM). MOXFQ has 3 separate underlying dimensions: pain, activity, and social interaction. The raw score of maximum 64 was converted to a metric index from 0 (best) to 100 (worst) (Morley et al. 2013). MOXFQ is available in Norwegian and is not validated for ankle fractures. The MOXFQ is validated for hallux valgus surgery and has been found to be highly responsive (Dawson et al. 2007). Other secondary measures were the Olerud–Molander Ankle (OMA) score (Olerud and Molander 1984), EuroQol-5D (EQ-5D) index, EQ-5D visual analogue scale (VAS), and VAS scores for pain during rest, during walking, at night, and during daily activities. OMA is a self-reported scale validated for ankle fractures, ranging from 0 (worst) to 100 (best). EQ-5D is a well-validated generic health-related quality-of-life instrument. Ankle range of motion was measured, comparing injured with non-injured ankle. The examination was standardized by a blinded physiotherapist, measuring dorsal and plantar flexion with a goniometer, with the foot placed on a 25 cm high foot stool with the knee in flexion.

### Radiological measurements

**Figure 2.**
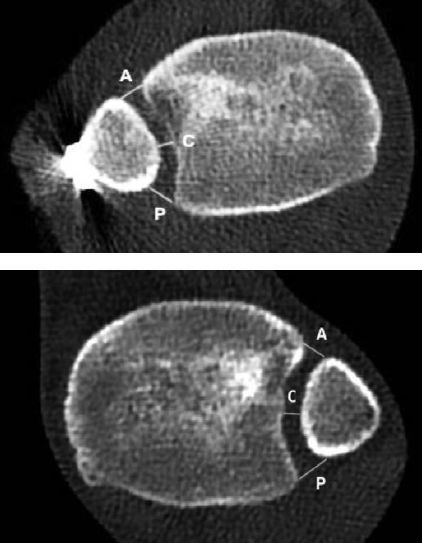
CT of injured ankle (upper panel) and uninjured ankle (lower panel) in a 20-year-old woman, 2 years after injury. Tibiofibular distance is measured on axial CT 1 cm proximal to the ankle joint. Distance measured anterior (A); central (C); and posterior (P).

Plain radiographs of the injured ankle were obtained after surgery, and at 6 weeks and 6 months. CT scans of both ankles were obtained postoperatively, and after 1 and 2 years. CT scans were standardized with the patient in a supine position, placing the feet in a purpose-made device, keeping the ankles in neutral position with 20° internal rotation of the legs. Radiological measurements were performed by 1 senior musculoskeletal radiologist (SBJ) and one orthopedic surgeon (BWR). The syndesmosis was assessed postoperatively and after 1 and 2 years by measuring the tibiofibular distance on axial CT scans, 1 cm proximal to the midpoint of the tibial plafond (Figure 2). The difference between injured and uninjured side was calculated. A criterion of < 2 mm difference in tibiofibular distance was selected for acceptable syndesmotic reduction (Andersen et al 2019, Patel et al. 2019). Signs of ankle osteoarthritis (OA), synostosis, talar exostoses, broken screws, and osteochondral lesions were reported. When assessing OA on CT scans, we defined mild OA as presence of osteophytes, and advanced OA as narrowing of the joint space and presence of cysts and sclerosis (Ray et al. 2019).

### Statistics

Sample size was calculated according to the equivalence criterion (Piaggio et al. 2012). The minimal clinically important difference (MCID) for ankle fracture patients is not defined for the AOFAS score but has been suggested to be half of the standard deviation (SD) (Norman et al. 2003). Based on data from previous trials with a similar population, the SD was estimated to 12 points (Wikeroy et al. 2010, Andersen et al. 2018), giving an MCID of the AOFAS score of 6 points. A between-group difference of 10 points (AOFAS) was used to ensure a sufficient inclusion of patients. 38 patients had to be included in each group to achieve a power of 0.95 and a significance level of 0.05. To strengthen the data and compensate for loss to follow-up, we planned to include 60 patients in each group. Analyses of endpoint results were performed as both intention-to-treat and per-protocol. Student’s T-test was used to compare means of normally distributed data. The Mann–Whitney U-test was used in cases of skewed data. Fisher’s exact test was used for categorial data. Data is reported as numbers, mean with SD, or median with interquartile range (IQR). We considered a probability of less than 5% as statistically significant and used 95% confidence intervals (CI) to describe uncertainty. Data analysis was conducted in IBM SPSS Statistics for Mac version 26 (IBM Corp, Armonk, NY, USA).

### Ethics, registration, funding, and potential conflicts of interest

Patients gave their written consent prior to randomization. The trial was conducted in accordance with the Declaration of Helsinki, approved by the National Committees for Research Ethics in Norway 2015/1860 and registered at ClinicalTrials.gov (NCT02930486). The study did not receive external funding. The authors have no conflicts of interest to declare.

## Results

## Results are reported according to the CONSORT guidelines

113 patients were randomized and allocated to SB (= 58) or TS (= 55) (Figure 1). The 2-year follow-up rate was 84%; the radiological follow-up rate was 81%. The baseline demographic patient characteristics and fracture treatment were reported (Table I).

**Table 1.** Patient characteristics at time of enrolment. Values are number of patients unless otherwise specified

Characteristic	SB (n = 55)	TS (n = 58)
Mean age (SD)	44 (15)	48 (14)
Male sex	35	30
Right side	32	26
Mean BMI (SD)	27 (5)	26 (4)
Medial malleolar fracture	14	19
Posterior malleolar fracture	37	31
Medial and posterior malleolar fracture	10	15
Maisonneuve fracture	26	20
Osteochondral damage of the talus	2 **^a^**	4
Intra-articular loose bodies	9 **^a^**	10
Temporary external fixator	7	2

**^a^**n = 54

### Clinical outcomes

**Figure 3.**
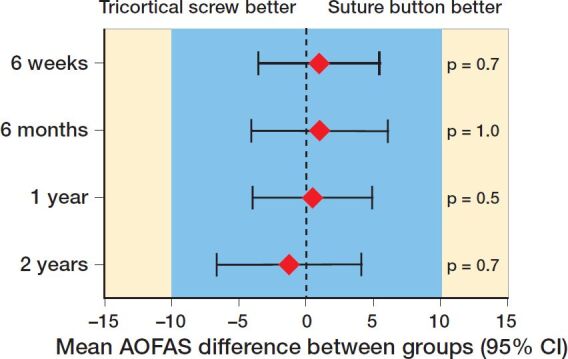
AOFAS equivalence diagram. Blue area indicates margins of equivalence defined as the between-group difference of 10 points. Results at all time intervals are equivalent since the 95% CI lies wholly inside the margins.

The groups did not differ statistically regarding clinical outcome: at 2 years, the median AOFAS score was 97 in both groups (IQR SB 87–100 vs. TS 90–100, p = 0.7) (Table 2). The difference in mean AOFAS was < 2, equivalent at all controls (Figure 3). Median MOXFQ was 5 in the SB group and 3 in the TS group (IQR SB 0–18 vs. TS 0–8, p = 0.2) (Table 2), and median OMA score was 90 in the SB group and 100 in the TS group (IQR SB 75–100 vs. TS 83–100, p = 0.2). Similarly, no statistically significant difference was detected in VAS, EQ-5D VAS, or EQ-5D (Table 2). Fracture pattern affected clinical outcome when we stratified the groups according to fracture pattern: after 2 years, patients with a Maisonneuve fracture pattern had better outcome scores with a median AOFAS at 100 in the Maisonneuve patients group compared with 95 in all other injuries (IQR 95–100 vs. 85–100, p = 0.001), while patients with trimalleolar fractures did worse, with a median AOFAS at 92 compared with 99 in other injuries (IQR 85–97 vs. 90–100, p = 0.03) (Table 3, see Supplementary data). The ability to plantar- and dorsiflex the ankle was similar between the groups. At 2 years, the mean difference between injured and uninjured ankle in plantar and dorsiflexion was ≤ 5° (Table 4, see Supplementary data). Per-protocol analyses supported the intention-to-treat findings.

**Table 2.** Primary and secondary outcome measures

Outcome measure	SB **^a^**		TS **^a^**		p-value
	n	score	n	score	
AOFAS					
6 weeks	54	67 (10)	52	66 (13)	0.7 **^c^**
6 months	53	87 (82–98)	54	88 (77–98)	1.0 **^b^**
1 years	53	93 (82–100)	52	90 (84–99)	0.5 **^b^**
2 years	48	97 (87–100)	47	97 (90–100)	0.7 **^b^**
MOXFQ					
6 weeks	52	29 ((11)	48	31 (13)	0.4 **^c^**
6 months	55	14 (3–31)	53	14 (3–36)	0.7 **^b^**
1 year	52	5 (0–32)	51	6 (0–13)	0.9 **^b^**
2 years	48	5 (0–18)	47	3 (0–8)	0.2 **^b^**
OMA					
1 year	53	90 (73–100)	52	90 (76–100)	0.4 **^b^**
2 years	47	90 (75–100)	45	100 (83–100)	0.2 **^b^**
VAS for pain during rest					
6 weeks	53	1.0 (0–2)	49	1.0 (0–2)	0.9 **^b^**
6 months	54	0.0 (0–1)	54	0.0 (0–2)	0.1 **^b^**
1 year	53	0.0 (0–1)	52	0.0 (0–1)	0.5 **^b^**
2 years	48	0.0 (0–1)	47	0.0 (0–0)	0.6 **^b^**
VAS for pain during walking					
6 weeks	53	2.0 (1–4)	49	3.0 (2–4)	0.3 **^b^**
6 months	54	1.0 (0–3)	54	1.0 (0–2)	0.8 **^b^**
1 year	53	1.0 (0–2)	52	1.0 (0–2)	0.9 **^b^**
2 years	48	0.0 (0–1)	47	0.0 (0–1)	0.2 **^b^**
VAS for pain at night					
6 weeks	53	1.0 (0–2)	49	1.0 (0–3)	0.6 **^b^**
6 months	54	0.0 (0–0)	54	0.0 (0–1)	0.01 **^b^**
1 year	53	0.0 (0–0)	52	0.0 (0–0)	1.0 **^b^**
2 years	48	0.0 (0–1)	47	0.0 (0–0)	0.2 **^b^**
VAS for pain during daily activity					
6 weeks	53	3.0 (2–6)	49	4.0 (2–7)	0.4 **^b^**
6 months	54	1.0 (0–3)	54	1.0 (0–2)	0.9 **^b^**
1 year	53	0.0 (0–2)	52	1.0 (0–2)	0.6 **^b^**
2 years	48	0.0 (0–29	47	0.0 (0–0)	0.03 **^b^**
EQ-5D index					
6 weeks	53	0.7 (0.6–0.8)	53	0.7 (0.3–0.7)	0.1 **^b^**
6 months	54	0.8 (0.7–1.0)	54	0.8 (0.7–1.0)	0.9 **^b^**
1 year	53	1.0 (0.8–1.0)	52	1.0 (0.8–1.0)	1.0 **^b^**
2 years	48	1.0 (0.8–1.0)	47	1.0 (0.9–1.0)	0.3 **^b^**
EQ-5D VAS					
6 weeks	52	73 (15)	51	63 (18)	0.004 **^c^**
6 months	53	89 (70–95)	54	80 (74–90)	0.2 **^b^**
1 year	52	85 (71–95)	52	88 (76–90)	0.6 **^b^**
2 year	48	85 (70–95)	45	90 (77–95)	0.6 **^b^**

**^a^** For not normally distributed data values are given as median (IQR)

in parentheses and for normally distributed data as mean (SD).

**^b^** Nonparametric (Mann–Whitney U) test.

**^c^** 2-sided t-test for independent samples.

**Table 5.** Radiological results: difference measured in mm in tibiofibular distance at level of syndesmosis (1 cm proximal to the ankle joint) between injured and uninjured side. Values are mean (SD) or median (IQR) unless otherwise specified

Factor	SB		TS		Mean between-group difference (95% CI)	p-value **^a^**
	n	difference	n	difference		
Difference in anterior distance						
≤ 2 weeks	54	0.1 (1.9)	56	0.7 (1.8)	–0.5 (–1.2 to 0.2)	0.1
1 year	54	1.1 (2.0)	50	0.7 (1.8)	0.3 (–0.4 to 1.1)	0.4
2 years	46	0.9 (1.9)	45	0.7 (1.6)	0.2 (–0.5 to 1.0)	0.5
Difference in central distance						
≤ 2 weeks	54	0.1 (1.2)	56	–0.7 (1.1)	0.2 (–0.2 to 0.6)	0.3
1 year	54	1.2 (1.9)	50	0.9 (1.4)	0.3 (–0.3 to 1.0)	0.3
2 years	46	1.4 (0.0–2.0)	45	1.0 (0.0–1.0)	0.7 (0.0 to 1.4)	0.2 **^b^**
Difference in posterior distance						
≤ 2 weeks	54	–0.4 (2.2)	56	–0.6 (2.1)	0.2 (–0.6 to 1.0)	0.7
1 year	54	0.1 (1.8)	50	0.4 (1.8)	–0.3 (–1.0 to 0.4)	0.5
2 years	46	0.0 (2.3)	45	0.3 (2.0)	–0.4 (–1.2 to 0.5)	0.4

**^a^**Levene’s test was used to assess equality of the variances. Statistical analysis was conducted using the 2-sided t-test for independent samples in normally distributed data;

otherwise the Mann–Whitney U-test was used.

**^b^**The Mann–Whitney U-test was used.

### Radiological results

At 2 years, 30 patients in the SB group and 27 patients in the TS group had radiological signs of ankle OA (RR 1.1, CI 0.7–1.7). When analyzing for advanced OA, there was a difference between the groups at 2 years: 8 patients in the SB group and 1 patient in the TS group had advanced OA (RR 8, CI 1–60). The groups displayed similar results when analyzing presence of talar osteophytes at 2 years: 12 in the SB group and 7 in the TS group (p = 0.3). At 2 years, 0 patients in the SB group and 5 patients in the TS group had complete synostosis (p = 0.03) (Figure 4, see Supplementary data). When stratifying the complete cohort at 2 years according to fracture pattern, patients with a Maisonneuve fracture had less OA (15 vs. 42, RR 0.7, CI 0.4–1.0), patients with a trimalleolar fracture had more OA (19 vs. 38, RR 1.6, CI 1.2–2.1).

The tibiofibular distance measured on CT scans postoperatively and after 1 and 2 years was similar between the groups. At 2 years, the mean difference in tibiofibular distance was ≤ 1 mm for anterior, central, and posterior measurement in both groups (Table 5). When applying a tibiofibular difference of < 2 mm between injured and uninjured ankle as a criterion for acceptable reduction the groups had similar results at all controls; 19 patients in the SB group and 16 patients in the TS group had an anterior difference > 2 mm postoperatively (RR 1.2, CI 0.7–2.1) (Table 6, see Supplementary data). After 2 years, 35 of 45 patients still had their TS implanted; 10 screws were broken.

### Complications and reoperations

10 patients in the SB group and 17 patients in the TS group had ≥ 1 reoperation (p = 0.2) (Table 7, see Supplementary data). 5 patients in the SB group and 11 patients in the TS group had their implants removed because of local irritation alone (p = 0.2). 3 patients in the SB group and 3 patients in the SS group required early reoperation (< 3 weeks) after CT postoperatively revealed unacceptable reduction of the fracture or of the syndesmosis (3 syndesmosis malreductions, 1 fibula malreduction, 2 medial malleolus malreduction). 2 patients (male, age 50 and female, age 52 years) suffered a low-energy tibia fracture through the suture button canal (Figures 5, 6, see Supplementary data). The male patient presented 6 months postoperatively with a healed tibia fracture with 13° varus deformity. Since this patient had no complaints the fracture was not addressed surgically. The female patient presented initially with a large posterior malleolar fracture. She presented with pain while walking 4 months after her initial injury. She had suffered a tibia fracture and was reoperated on with open reduction and internal fixation. A dual energy X-ray absorptiometry (DEXA scan) showed osteoporosis.

## Discussion

The main findings in this study are equivalent clinical results in patients treated with either an SB or an TS 2 years after acute syndesmotic injury. The mean AOFAS difference between the groups was overlapping and inside the margins of the 95% CI at all controls. The rate of appropriate syndesmotic reduction, reoperations, and rate of OA was similar between our groups. In the SB group, 2 patients experienced fractures through the suture button canal. 5 patients in the TS group had synostosis after 2 years. Fracture pattern affected clinical outcome.

The clinical results are in contrast to earlier studies reporting better results for SB fixation (Shimozono et al. 2018). An explanation for this discrepancy could be the different mechanical properties between the screw options for fixation. The dynamic properties of syndesmotic implants in vivo are unknown, but there are mechanical studies on the subject. Fixation of the syndesmosis with several 3.5 mm tricortical SS or a 4.5 mm quadricortical SS locks the fibula in the incisura, while the TS has in a cadaver study displayed more dynamic properties (Clanton et al. 2017). This may explain why Andersen et al. (2018) found a quadricortical SS to be inferior to an SB, while Kortekangas et al. (2015) found no difference when comparing an TS with an SB.

The first SBs available required a suture knot on the lateral side, with irritation and a reported removal rate of 6% (Andersen et al. 2018). We used a knotless SB to potentially lower this rate. Despite this, our removal rate was 9%. Changing to a knotless SB did not affect the removal rate. This could be due to other factors, such as irritation from the fibula plate, present in almost half of the SB patients. 6 patients required early reoperation, based on postoperative CT scans. We advocate a low threshold for obtaining postoperative CT scans after syndesmotic reduction (Garner et al. 2015, Barbosa et al. 2020).

Trauma is the most common cause of ankle OA (Saltzman et al. 2005). The rate of radiologic OA after 2 years was high in this study. The reason for this could be the use of CT, which is more sensitive than radiographs when assessing OA. Most of the patients (48 of 57) displayed only minor signs of OA. The rate of advanced OA in 9 patients is in line with previous studies (Lübbeke et al. 2012, Ray et al. 2019). The observation period of 2 years is short and the study population is underpowered to conclude on the differences in advanced OA between the groups. More patients had complete synostosis in the TS group, supporting the findings by Hinds et al. (2014) that SS fixation is a risk factor for synostosis development. 2 patients treated with SB suffered a non-traumatic fracture through the suture button canal. This specific complication and its incidence have not been reported in the literature. We suggest a syndesmotic screw as a better alternative in patients with poor bone quality.

A weakness in the study is our choice of outcome score. The ideal outcome score should be validated for the injury in question, have high reliability, and be available in the language of the patients examined. Our primary outcome, the AOFAS, is not validated; it is criticized for low precision, and for producing skewed data due to ceiling effects (Veltman et al. 2017). Even so, the AOFAS was chosen because of its widespread use. We decided to add the MOXFQ, since it was available in Norwegian. It is validated for hallux valgus surgery, not ankle fractures, hence its properties for ankle fractures are not known. After initiation of our trial, a comparison of 3 different PROMs available in Norwegian were published, recommending the Self-Reported Foot and Ankle Score (SEFAS) for evaluating patients with ankle fractures (Garratt et al. 2018). Another weakness is the lack of standardization in the syndesmosis fixation and several surgeons treating the patients. This could be a source of uncontrolled variability between the groups. On the other hand, it makes our results transferable to the day-to-day practice of fracture surgery.

The primary strengths of this study are the randomized prospective design with blinded scoring of clinical outcome measures, comparable groups at baseline, a high follow-up rate, and CT evaluation 2 years after treatment. In addition, all hospitals participating in the study used both implants as standard treatments before initiation of the trial, minimizing problems with the learning curve associated with new treatments. The procedure was performed by the on-call team, providing generalizability. Our outcome scores after 2 years are in line with scores from similar studies (Wikeroy et al. 2010, Laflamme et al. 2015, Andersen et al. 2018), supporting previous data on outcomes after syndesmotic injury.

### Interpretation

In this RCT comparing a knotless SB and an TS we found no clinically relevant differences regarding outcome scores between the groups. TS is an inexpensive alternative to SB when treating acute syndesmotic injury.

## Supplementary Material

Supplemental Material

## Supplementary data

Tables 3, 4, 6, 7 and Figures 4–6 are available as supplementary data in the online version of this article, http://dx.doi.org/10.1080/17453674.2020.1818175
