# Supplementary material for: Randomized trial comparing suture button with single 3.5 mm syndesmotic screw for ankle syndesmosis injury: similar results at 2 years
Source: Acta Orthop. 2020 Sep 10;91(6):770–5. doi: 10.1080/17453674.2020.1818175 (PMC8023901; doi:10.1080/17453674.2020.1818175)
Supplement: Supplemental Material [file IORT_A_1818175_SM3023.pdf]

Supplementary data

Table 3. Clinical outcome scores according to fracture patterns

Factor	Follow-up year	Specified fracture	Other fractures	p-value
**Maisonneuve fracture (n = 31) versus other fractures (n = 58)**				
AOFAS	1	100 (83–100)	90 (81–97)	0.01
	2	100 (95–100)	95 (85–100)	0.001
MOXFQ	1	3 (0–13)	8 (1–27)	0.02
	2	0 (0–3)	4 (0–11)	0.01
OMA	1	90 (85–100)	88 (70–100)	0.03
	2	100 (85–100)	90 (79–100)	0.07
**Trimalleolar fracture (n = 23) versus other fractures (n = 66)**				
AOFAS	1	87 (80–95)	93 (85–100)	0.02
	2	92 (85–97)	99 (90–100)	0.03
MOXFQ	1	11 (2–27)	5 (0–16)	0.2
	2	5 (1–11)	2 (0–6)	0.03
OMA	1	80 (70–95)	90 (80–100)	0.03
	2	90 (80–95)	98 (80–100)	0.06

Values are given as median (IQR). Statistical analysis was conducted using nonparametric (Mann–Whitney U) test.

Table 4. Ankle range of movement. Values are number and mean (SD) difference in degrees between injured and uninjured ankle

Factor	SB n	SB difference	TS n	TS difference	p-value
6 months					
plantar flexion	46	8 (10)	39	10 (10)	0.4
dorsiflexion	46	11 (8)	39	10 (8)	0.6
1 year					
plantar flexion	46	6 (10)	38	7 (9)	0.4
dorsiflexion	46	5 (6)	38	5 (6)	0.7
2 years					
plantar flexion	41	4 (7)	34	5 (9)	0.5
dorsiflexion	42	5 (6)	34	4 (5)	0.3

Statistical analysis was conducted using 2-sided t-test for independent samples.

Table 6. Malreduction: number of patients with ≥ 2 mm difference in tibiofibular distance between injured and uninjured ankle of all examined patients with relative risk (RR) estimates

Factor	SB	TS	RR (95% CI)	p-value
Difference in anterior distance				
≥ 2 weeks	19/54	16/56	1.2 (0.7–2.1)	0.5
1 year	21/54	18/50	1.1 (0.7–1.8)	0.8
2 years	19/46	13/45	1.4 (0.8–2.5)	0.3
Difference in central distance				
≥ 2 weeks	7/54	9/56	0.8 (0.3–2.0)	0.8
1 year	18/54	11/50	1.5 (0.8–2.8)	0.3
2 years	16/46	9/45	1.7 (0.9–3.5)	0.2
Difference in posterior distance				
≥ 2 weeks	26/54	26/56	1.0 (0.7–1.5)	1.0
1 year	14/54	14/50	0.9 (0.5–1.7)	0.8
2 years	22/46	15/45	1.4 (0.9–2.4)	0.2

Statistical analysis was conducted using Fisher's exact test.

Table 7. Reoperations. Values are number of patients

Primary complaint/indication	Reoperation type	SB	TS
Early reoperations (< 3 weeks) Unacceptable fracture or syndesmotic reduction	Refixation < 3 weeks	3	3
Fracture in SB canal	Revision and refixation	1	0
Deep infection	Operative debridement	1	1
Recurrent syndesmotic diastasis	Revision repair	1	0
Implant irritation(> 9 months)	Implant removal	4	11
Screw migration with risk of skin penetration	Implant removal	0	1
Screw breakage and recurrent syndesmotic diastasis	Revision repair	0	1
Total		10	17

 Acta Orthopaedica 2020; 91 (DOI: 10.1080/17453674.2020.1818175)

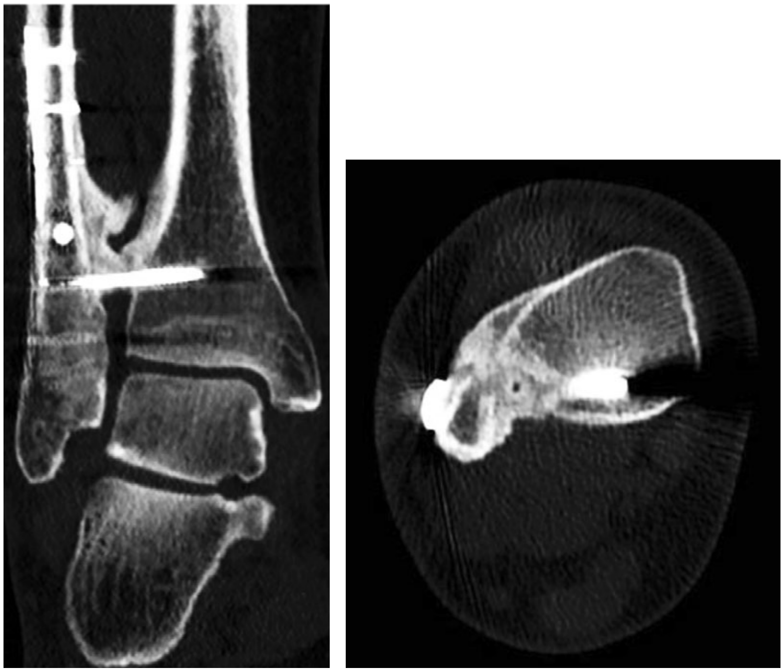

Figure 4. CT of 65-year-old man, 2 years after injury. Coronal and axial views of a complete tibiofibular synostosis.

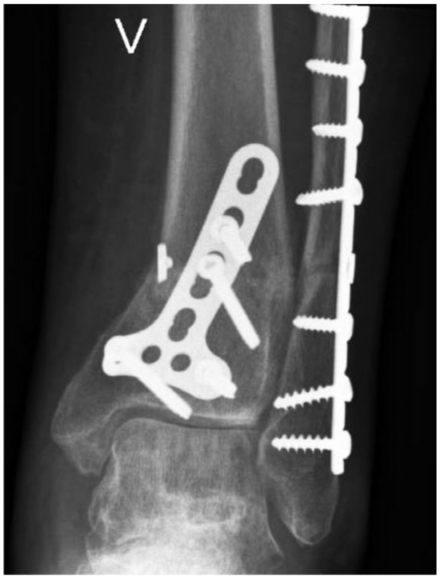

Figure 5. 52-year-old woman, fracture through the suture button canal, 4 months after initial injury.

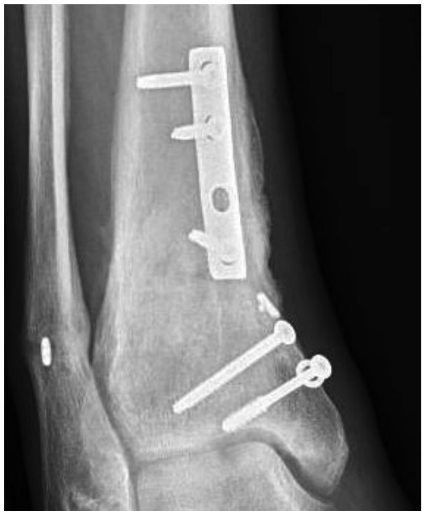

Figure 6. 50-year-old man with a healed fracture through the suture button canal, 6 months after initial injury.
